# Synthesis of Carvacrol-Loaded Invasomes Nanoparticles Improved Acaricide Efficacy, Cuticle Invasion and Inhibition of Acetylcholinestrase against Hard Ticks

**DOI:** 10.3390/microorganisms11030733

**Published:** 2023-03-13

**Authors:** Amr Gamal, Shawky M. Aboelhadid, Fatma I. Abo El-Ela, Abdel-Azeem S. Abdel-Baki, Samar M. Ibrahium, Almahy M. EL-Mallah, Saleh Al-Quraishy, Ahmed O. Hassan, Sahar M. Gadelhaq

**Affiliations:** 1 Department of Pharmaceutics and Industrial Pharmacy, Faculty of Pharmacy, Beni-Suef University, Beni-Suef 62511, Egypt; 2 Parasitology Department, Faculty of Veterinary Medicine, Beni-Suef University, Beni-Suef 62511, Egypt; 3 Department of Pharmacology, Faculty of Veterinary Medicine, Beni-Suef University, Beni-Suef 62511, Egypt; 4 Zoology Department, Faculty of Science, Beni-Suef University, Beni-Suef 62521, Egypt; 5 Department of Parasitology, Animal Health Research Institute, Fayum Branch, Fayum 16101, Egypt; 6 Zoology Department, College of Science, King Saud University, Riyadh P.O. Box 2455, Saudi Arabia; 7 Department of Medicine, Washington University School of Medicine, St. Louis, MO 63110, USA; 8 Parasitology Department, Faculty of Veterinary Medicine, Minia University, Minia 61519, Egypt

**Keywords:** carvacrol, invasomes, nanoparticles, acaricides, repellent, HPLC, acetylcholinesterase

## Abstract

Carvacrol is a monoterpenoid phenol found in many essential oils that has antibacterial, antifungal and antiparasitic activities. Drug loaded-invasome systems are used to deliver drugs utilizing nanoparticles to improve bioavailability, efficacy, and drug release duration. As a result, the present study developed carvacrol-loaded invasomes and evaluated their acaricidal effect against *Rhipicephalus annulatus* (cattle tick) and *Rhipicephalus sanguineus* (dog tick). Carvacrol loaded-invasome (CLI) was prepared and characterized using UV/Vis spectrophotometer, zeta potential measurements, Scanning Transmission Electron Microscopy (STEM), Fourier Transform Infrared (FT-IR) Spectroscopy, and Differential Scanning Calorimetry Analysis. CLI (5%) induced significant mortality (100%) in *R. annulatus* adult ticks with LC_50_ of 2.60%, whereas the LC_50_ of pure carvacrol was 4.30%. Carvacrol and CLI were shown to have a significant larvicidal action on both tick species, with LC_50_s of 0.24 and 0.21% against *R. annulatus* and 0.27 and 0.23% against *R. sanguineus*, respectively. Carvacrol and CLI (5%) induced significant repellent activities for 24 h against *R. annulatus* and *R. sanguineus*, as evidenced by the rod method and the petri-dish selective area choice method, respectively. High-performance liquid chromatography (HPLC) demonstrated that the CLI form had 3.86 times the permeability of pure carvacrol. Moreover, carvacrol and CLI inhibited acetylcholinesterase activity and decreased glutathione and malonedealdehyde levels in the treated ticks. In conclusion, invasomes significantly improved adulticidal and repellency activities of carvacrol against both tick species.

## 1. Introduction

Ticks are hematophagous arthropods that parasitize the majority of vertebrate species worldwide, including human and animals [1]. Egypt is not an exception, and ticks cause significant economic losses both directly by blood sucking and indirectly by acting as vectors for various pathogens [2]. Tick bites also lower the quality of hides, and feeding by huge numbers of ticks reduces the live weight gain and induces anemia among domestic animals [3]. The potential of ticks to transmit protozoan, rickettsial, and viral of diseases of livestock, which are of enormous economic importance worldwide, causes the majority of tick-related losses [4]. According the latest review of hard ticks in Egypt, 52.5% of dogs were infested with the *Rhipicephalus sanguineus* tick and 50% of cattle were infested with the *Rhipicephalus annulatus* tick [2,5]. Abdelbaset et al. [4] also proved the circulation of zoonotic tick-borne pathogens among dogs, cattle, and tick vectors in Egypt. Therefore, intensive tick control is urgently required. The most common method of tick control in Egypt, as in many other countries, is the use of synthetic acaricides. However, widespread acaricidal resistance, unavailability, and high acaricide costs, particularly for low-income farmers in developing countries, highlight the need for alternate tick control approaches [6]. Several plant extracts, plant essential oils, and their chemical components are a promising option for discovering such alternatives [7].

Carvacrol is a volatile phenolic monoterpene found predominantly in essential oils extracted from plants of the genus *Lippia* (Verbenaceae) [8]. Much research has demonstrated that carvacrol has antioxidative, anti-inflammatory, antibacterial, antiviral, antifungal, antiprotozoal, anticarcinogenic, antidiabetic, and neuroprotective activities [9]. They attributed these actions to hydrophobic properties associated with the substituted aromatic ring and the hydrophilic characteristics of the phenolic OH group [9,10,11]. In addition, carvacrol showed strong acaricidal activity against *Amblyomma americanum, Hyalomma marginatum, Rhipicephalus turanicus*, *R. sanguineus s.l.*, and *R. microplus* [12,13,14,15,16,17]. In addition, carvacrol and thymol impaired the oxidative balance in *R. microplus* larvae through increasing the activities of the glutathione-S-transferase (GST), catalase (CAT), superoxide dismutase (SOD), and glutathione peroxidase (GPX) at different lethal concentrations [18]. However, the high volatility at room temperature, along with an almost total lack of idrosolubility and low diffusion rate, limited the use of carvacrol as an acaricidal agent. In order to solve this limitation, in recent years, different types of nanocarriers have been designed [19]. Among these nanocarriers, invasomes, which are vesicular systems with phospholipids, ethanol, and terpenes in their structure, appear to be suitable carriers [20]. These invasomes entrap the volatile compounds and improve their stability, solubility, and transdermal penetration [19,20,21].

Following this line of thought, a carvacrol-loaded invasome (CLI) was synthesized and tested against two important species of ticks: *Rhipicephalus sanguineus* (brown dog tick) and *Rhipicephalus annulatus* (cattle tick).

## 2. Material and Methods

### 2.1. Materials

Cineole, ethanol, phospholipid, and cholesterol were purchased from Agitech Pharmaceutical Company (Cairo, Egypt). Methanol and chloroform were obtained from Cornell Lab in Egypt. Carvacrol was purchased as a pure compound from Sigma Aldrich, Darmstadt, Germany. The pure carvacrol 5% was prepared in 2% of tween 80 to use in the applications in this work.

### 2.2. Preparation of Carvacrol-Loaded Invasome (CLI)

A carvacrol-loaded invasomes (CLI) formulation was prepared by a thin hydration method, as described by Shah et al. [22]. The calculated amounts of carvacrol (10 mg), cineole (1% *v*/*v*), cholesterol (0.15% *w*/*w*), and phospholipid (3% *w*/*w*) were dissolved in an organic solution (10 mL) of chloroform and methanol (3:1). This solution was poured in a conical flask and then evaporated under vacuum using a Stuart rotary evaporator (RE300, Mainland, UK) at 100 revolutions per minute at 40 °C. After evaporation, a thin film of invasomes formed inside the flask. At 40 °C for 1 h, isotonic phosphate buffer (IPB, pH 5.5) solution and ethanol (3% *v*/*v*) solution were added to hydrate the generated lipid film. The prepared carvacrol-loaded invasome (CLI) formulation was sonicated and kept at 4 °C.

### 2.3. In Vitro Evaluation of Carvacrol-Loaded Invasome (CLI) Formulation

#### Entrapment Efficiency (EE%) Measurement

A standard calibration curve was constructed using a UV/Vis spectrophotometer at 277 nm to detect the amount of carvacrol in an unknown sample [23]. The percentage of entrapment efficiency (EE) was used to calculate the amount of carvacrol entrapped in the CLI formulation (Equation (1)) [24]. Carvacrol content was evaluated by centrifuging a sample of the produced CLI formulation at 20,000 rpm for 1 h. The CLI pellets were isolated, and the amount of carvacrol in the supernatant was quantified in three replicates using a UV/Vis spectrophotometer at 277 nm [25].

EE% = ((Initial carvacrol amount − The amount of carvacrol in the supernatant))/(Initial carvacrol amount) × 100. (1)

### 2.4. Vesicle Size and Zeta Potential Measurement

Dynamic Light Scattering (DLS) analysis was performed using a Zetasizer (Malvern, Herrenberg, Germany) to assess the vesicle size, polydispersity index (PDI), and zeta potential of CLI [26]. The particle size and polydispersity index (PDI) were analyzed to measure the particle’s dispersion, homogeneity, distribution, and subsequent targeting ability of the CLI formulation [26]. The electrostatic charge and stability of the CLI formulation were evaluated using the zeta potential [26]. Briefly, 1 mL of CLI formulation was diluted with 9 mL of distilled water and measured three times using dynamic light scattering to quantify particle size, PDI, and zeta potential (DLS, Malvern, Germany).

### 2.5. Scanning Transmission Electron Microscopy (STEM) Investigation

Scanning transmission electron microscopy (STEM) Carl Zeiss, Oberkochen, Germany) was used to investigate the morphology of the CLI formulation and its surface properties [24]. A sample of the CLI formulation was deposited on a carbon-coated copper grid and visualized using STEM at suitable magnifications [27].

### 2.6. Fourier Transform Infrared (FT-IR) Spectroscopy

The chemical interactions and compatibility of carvacrol with the components of the optimal CLI formulation were evaluated using FTIR (8400s, Shimadzu, Tokyo, Japan) [28]. The samples were thoroughly pulverised and mixed with KBr before being analyzed from 4000 to 400 cm^−1^.

### 2.7. Differential Scanning Calorimetry Analysis

A differential scanning calorimetry (DSC) analysis was obtained using a calorimeter (NETZSCH-Geratebau GmbH, Maia, Germany) to characterize the thermal analysis of carvacrol and individual components of optimum CLI formulation [28]. The DSC analysis was carried out to ascertain the melting point, the compatibility of the vesicle components, and the degree to which the enthalpy of a material had changed over time due to changes in its physical and chemical properties [28]. DSC thermograms were performed with a nitrogen flow rate of 25 mL/min and a heating rate of 5 °C/min. The samples were promptly cooled to 25 °C after being heated to 250 °C.

### 2.8. Preparation of R. annulatus and R. sanguineus Ticks Larvae

Females of *R. annulatus* and *R. sanguineus* ticks were collected from naturally infested cattle and dogs, respectively, in Beni-Suef Governorate, Egypt. This collecting district (Beni-Suef city, south of Cairo) suffered from tick control failure with the typical acaricide in this area (deltamethrin 5%). Ticks were obtained from cattle and dogs that had a history of tick infestations and had not been treated for at least a month. The collected ticks were transported to the Parasitology Laboratory at Beni-Suef University’s Faculty of Veterinary Medicine. The tick species verification was performed according to Estrada-Pea et al. [29]. A part of the collected ticks was placed in petri dishes with 10 ticks in each. They were subsequently used for the adult immersion bioassay. The other part of ticks was kept in in a BOD incubator for oviposition. Eggs were collected, mixed, and separated into 50 mg lots for testing.

### 2.9. Adult Immersion Test (AIT) for R. annulatus Tick

Carvacrol and carvacrol-loaded invasomes (CLI) were investigated for acaricidal activity against adult ticks, using the method of Drummond et al. [30]. Female ticks were immersed in tubes containing 10 mL of diluted carvacrol or CLI for 2 min at concentrations: 5, 2.5, 1.25, and 0.625%. Then, they were dried and incubated in petri dishes at 26–28 °C and 80% relative humidity. The pure carvacrol was dissolved in ethyl alcohol 70% while CLI was diluted by distilled water. For each concentration, five replicates of ten ticks were performed. Ticks in the negative control group were immersed in ethyl alcohol 70% or distilled water for 2 min, while the positive control group was treated with 1 mL/L Chlorpyrifos. The effectiveness of the application was assessed by counting the number of dead ticks after two weeks and calculating the egg production index for live ticks [31]:

$$\mathrm{EPI}=\frac{\mathrm{weight}\;\mathrm{of}\;\mathrm{egg}\;\mathrm{mass}}{\mathrm{initial}\;\mathrm{weight}\;\mathrm{of}\;\mathrm{engorged}\;\mathrm{female}}\times 100\;$$

### 2.10. Larvicidal Activity against R. annulatus and R. sanguineus Ticks

The larvicidal activity of carvacrol and CLI was evaluated using a modified larval packet technique (LPT) [32] at concentrations: 5, 2.5, 1.25, and 0.625%. Using a fine-tipped paintbrush, about 100 larvae were distributed across filter papers (7 × 7 cm). Then, 100 µL of each concentration was added. The treated filter papers were packed into packets. Ticks in the negative control group were immersed in ethyl alcohol 70%, while the positive control group was treated with 1 mL/L Chlorpyrifos. Each concentration was completed in 5 replicates. After 24 h, the treated packets were examined to determine mortality rates by counting live and dead larvae (motionless larvae were considered dead).

### 2.11. Repellency Activity against R. annulatus Larvae

The repellent activity of carvacrol and CLI was measured using a technique established by Wanzala et al. [33] based on the vertical migratory behavior of tick larvae. The apparatus used consisted of metal rods, each 23 cm long and 0.7 cm in diameter, mounted vertically in an aluminum base. A filter paper (6 cm in diameter) was treated with 180 µL of carvacrol or CLI (5%), while another rod received a filter paper treated with DEET 7% as a positive control and an untreated filter paper was stapled to another rod as a negative control. A total of 100 *R. annulatus* larvae (7–14 days old) were placed at the base of each rod and monitored for 15 min to check if they climbed up the rod in the first hour. They were then measured at the start of each hour, until 24 h had passed. The repellency percentage was calculated for each treatment by using the following formula:

$$\mathrm{Repellecy}\;\mathrm{perecentage}=\frac{\mathrm{Nt}-\mathrm{Nc}}{\mathrm{Nt}+\mathrm{Nc}}\times 100$$

where Nt and Nc are the numbers of larvae that climbed the treated and control rods, respectively [33].

### 2.12. Repellency Test against R. sanguineus Adult Tick

The choice test (repellent vs. untreated surface, tested material vs. DEET standard repellent as positive control) was conducted according to the approach given by Bissinger and Roe [34] and adapted by Ferreira et al. [35]. A filter paper, circular in shape, was placed in a Petri dish was divided into two equal halves. One half of the filter paper was treated with 200 µL of 5% of carvacrol, CLI, or 7% DEET. The other half of the filter paper was treated by the solvent (2% DMSO). The treated filter papers were allowed to air dry for 30 min before being used for testing. The repellent activity was also tested after 1, 2, and 4 h of drying time. To begin the test, five ticks (two males and three females) were placed in the center of the Petri dish in the absence of light in a temperature-controlled environment (26 ± 1 °C) with 70% relative humidity. The position of the tick was evaluated after 5 min. Each treatment was done in five replicates. The ticks in each application were not used again. The repellency % was estimated according to Ferreira et al. [35].

### 2.13. High-Performance Liquid Chromatography (HPLC) Chromatographic Investigation

The in vivo tick’s cuticle penetration of carvacrol-loaded invasomes (CLI) formulation was examined and compared to free carvacrol to evaluate the enhancing effect of invasomes on carvacrol’s permeation. Ticks were divided into three groups, with G1 serving as the negative control (2% tween 80) and G2 and G3 receiving free carvacrol (24 mg) and CLI formulation (equivalent to 24 mg carvacrol), respectively. Waters 2690 Alliance HPLC system equipped with a Waters 996 photodiode array detector was used to determine the concentration of carvacrol within the treated ticks. Carvacrol was isocratically separated using an analytical column C-18 with dimensions of 150 × 4.6 mm and a 50:50 *v*/*v* mobile phase composed of orthophosphoric acid and acetonitrile buffer solution. Carvacrol was detected at 275 nm using a mobile phase flow rate of 1 mL/min and a 10 µL injection volume. The linearity was obtained with R2 = 0.997 and a retention time of 8.59 min. Samples from each tick group were mixed with acetonitrile before being centrifuged for 10 min at 3.0× *g*. The supernatant was evaporated and dissolved in the mobile phase before being analyzed in triplicate by HPLC to determine the total amount of carvacrol that penetrated the tick’s cuticle.

Individual ticks from the control and treatment groups were processed and evaluated. The ticks were cleaned with distilled water and stored at −20 °C in 1 mL of acetonitrile. Each tick was crushed in 1 mL of acetonitrile (the original storage volume) with a glass pestle, agitated for 15 min, then centrifuged at 13,000× *g* for 10 min. The concentration of carvacrol was determined using a slightly modified version of the approach described by Mir’o et al. [36] for thymol. A volume of 500 μL of supernatant was diluted with a volume of 500 μL of ultrapure water. Then, 50 μL of the dilution was fed into a Shimadzu 10 HPLC system (Shimadzu Corporation, Kyoto, Japan) equipped with a Kromasil C18 reverse phase column (150 4.6 mm with a 50:50 *v*/*v* mobile phase) and a UV detector (Shimadzu, SPD-10A UV detector) reading at 274 nm. The mobile phase was composed of ultra-pure water (A) and acetonitrile (B) at a ratio of 47/53 at a flow rate of 1.5 mL/min.

The chromatographic peak regions of each analyte for carvacrol in normal or invasomes were determined using the integrator software (LC Solution, Shimadzu Corporation, 2695LC) of the HPLC system. The analytical techniques for measuring carvacrol in the tissues of *R. annulatus* (ticks) engorged females were validated before beginning the analysis of the experimental samples.

In brief, known amounts of each analyte were added to aliquots of untreated tick extracts to provide calibration standards (carvacrol: 24 μg/mL and 400 μg/mL), which were then evaluated by HPLC in triplicate. To all samples, 24 μL of carvacrol solution (4.2 μg/L) was added as an internal standard. Calibration curves for carvacrol with concentrations ranging from 0.1 to 24 μg/mL were also prepared as mobile phase standards. Calibration curves were generated using least squares linear regression analysis of analyte peak area ratios over the internal or external standard (carvacrol). The square correlation coefficients (R2) were close to one. The concentrations in the experimental samples were estimated by interpolating peak area ratios of the analytes on the external standard calibration line.

The mean standard deviation (SD) was used to indicate the concentration levels of each analyte in normal or invasomes. Each sample’s peak area was calculated and compared to the peak area of carvacrol at a concentration of 24 mg. The statistical analysis was carried out using the Student’s *t*-test (*p* < 0.001). The Student’s *t*-test was performed to compare the average levels of carvacrol analytes in engorged females from various groups with a *p*-value less than 0.05, indicating statistical significance. All statistical analyses were carried out using SPSS software (IBM SPSS Version 23).

### 2.14. Oxidative Stress and Acetylcholinesterase Inhibition in Treated Ticks

The ticks were macerated in mortar and suspended in lysis buffer (5 ticks for each treatment; pure carvacrol, CLI, Chlorpyrifos, and untreated ticks). In the presence of ice, the lysate materials were homogenized with a glass homogenizer. The tick homogenates were spun for 10 min at 10,000 rpm in a cooling centrifuge. The supernatants were then aspirated using a micropipette and preserved for use in the subsequent assays [37]. Ellman et al.’s [38] method was used to evaluate AchE activity in supernatants of treated ticks, and absorbance was measured at 412 nm. The percent of AchE inhibition was calculated according to Anderson and Coats [39] as the following:

$$\mathrm{AchE}\;\mathrm{inhibition}\;(\%)=100-[\frac{\mathrm{As}}{\mathrm{Ac}}\;\times \;100],\;$$

where: As = AChE activity in the treated ticks for each concentration; Ac = AChE activity in the untreated ticks.

Regarding the oxidative stress, glutathione (GSH) level was assessed according to Beutler et al. [40], whereas the lipid peroxidation (malondialdehyde MDA) was estimated according to Bar-Or et al. [41].

### 2.15. Statistical Analysis

The results of the different treatments were statistically analyzed using IBM SPSS for Windows, v.22 (IBM, Armonk, NY, USA). ANOVA was performed to analyze the differences between treatments, and Duncan’s tests were employed to estimate the differences between means (α = 0.05). SPSS v.22 was used to calculate the lethal concentrations, as well as the 50% and 90% mortality rates.

## 3. Results

### 3.1. Characterization of CLI Formulation

#### Entrapment Efficiency Measurement

The standard calibration curve was shown to be reliable for quantifying carvacrol with a coefficient of determination (R2) of 0.999, suggesting linearity. When the EE% of the CLI formulation was calculated, it was found to be 90.24 ± 0.92%.

### 3.2. Vesicle Size and Zeta Potential Measurement

**Figure 1:**
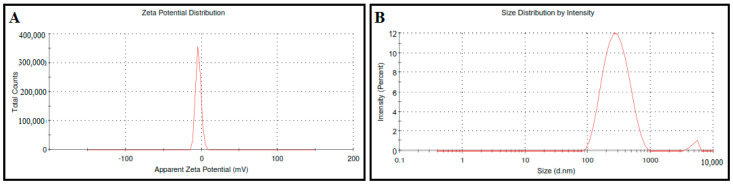
The zeta potential (**A**) and particle size (**B**) of carvacrol-loaded invasome (CLI) formulation.

The zeta potential and particle size of the CLI formulation are shown in Figure 1, which are −4.37 ± 0.52 mV and 267 ± 2.25 nm, respectively, with a low polydispersity index of 0.230 ± 0.05. The polydispersity index analysis demonstrated a low polydispersity index, indicating the presence of homogeneity and a narrow particle size distribution. The negative surface charge of the CLI formulation indicated high physical and chemical stability, sufficient for electrostatic stabilization.

### 3.3. Scanning Transmission Electron Microscopy (STEM) of Carvacrol-Loaded Invasome

**Figure 2:**
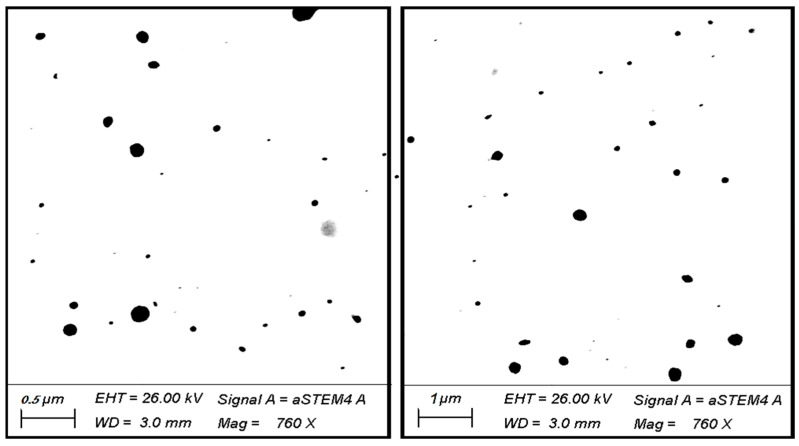
STEM of carvacrol-loaded invasome formulation. The black dots indicate the spherical shape of invasomes vesicles.

STEM micrographs of the surface morphology of the CLI formulation are shown in Figure 2. The CLI formulation was depicted in the STEM photos as spherical nano-vesicles with black dots.

### 3.4. Fourier Transform Infrared (FT-IR) Spectroscopy

**Figure 3:**
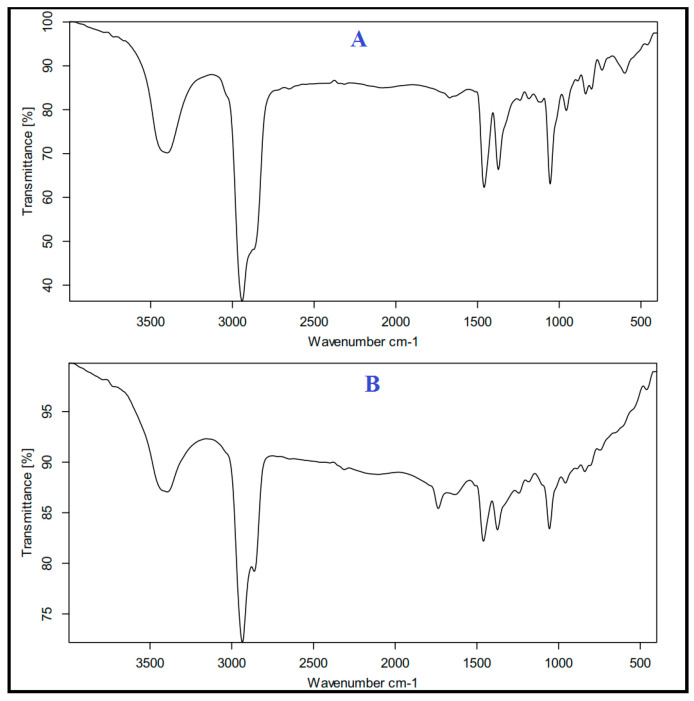
FTIR of Carvacrol (**A**) and CLI formulation (**B**).

Figure 3 shows the FTIR spectra of carvacrol (a) and optimum CLI formulation (b). The study of the FTIR spectrum of the carvacrol (Figure 3a) shows peaks bond at 1500, 2850 and 3360 cm^−1^, which are related to the stretching vibrations of C=C, C-H and –OH, respectively. The study of the FTIR spectrum of the optimum CLI formulation (Figure 3b) showed similar peaks as that of carvacrol, demonstrating compatibility in the formulation The spectrum confirms the well interaction of carvacrol with the phospholipids of the nano-invasome chemical structure.

### 3.5. Differential Scanning Calorimetry Analysis

**Figure 4:**
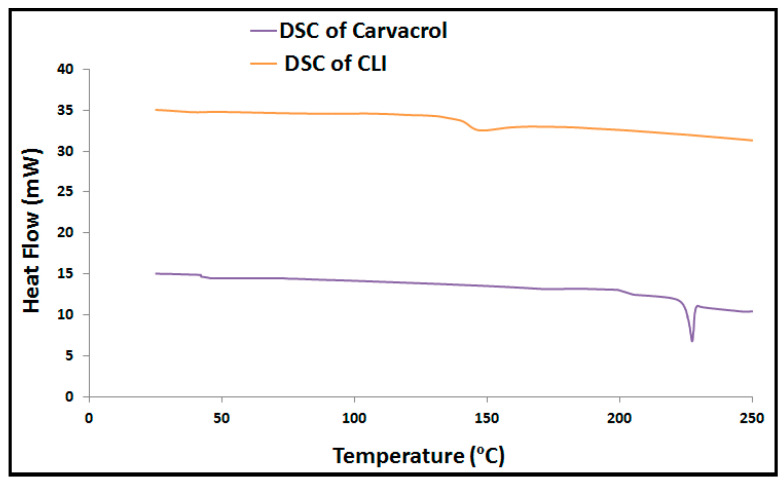
Thermal analysis of carvacrol and CLI formulations.

Figure 4 shows the endothermic peaks of carvacrol and optimum CLI formulation. The study of the DSC thermogram of the carvacrol shows endothermic peak at 241.17 °C, corresponding to its boiling point. Examination of the DSC thermogram of the CLI formulation reveals disappearance of the characteristic peak of carvacrol, indicating molecular encapsulation of the carvacrol inside the vesicles.

### 3.6. Adulticidal Activity against R. annulatus Ticks

The toxicity of carvacrol and CLI to *R. annulatus* adult ticks is concentration dependent. The concentration of 5% caused considerable mortality in adult *R. annulatus*. CLI caused substantial toxicity to the ticks, resulting in 100% death as compared to the pure form, which caused 62.0% mortality at 5% concentration. The LC50 and LC90 for carvacrol and CLI were reached at concentrations of 4.3 and 6.31% vs. 2.60 and 3.84%, respectively (Table 1). The egg production index (EPI) was assessed for the treated ticks, and the results revealed a significant zero percentage of EPI for ticks treated with CLI, while it was 42% for ticks treated with pure carvacrol.

### 3.7. Larvicidal Activity against R. annulatus and R. sanguineus Larvae

Carvacrol and CLI application resulted in a high significant larval mortality percentage (100%) for both larval tick species (*R. annulatus* and *R. sanguineus*), even at low concentrations (0.625%) (Table 2). The LC_50_ for carvacrol and CLI against *R. annulatus* larvae were reached at concentrations of 0.24% and 0.21%, respectively; they were at concentrations of 0.27% and 0.23% against *R. sanguineus* larvae. The invasome form of carvacrol had a lower numerical LC_50_ than the carvacrol pure form against both tick larvae species (Table 2).

### 3.8. Repellency Activity of Carvacrol-Loaded Invasome against R. annulatus Larvae

**Figure 5:**
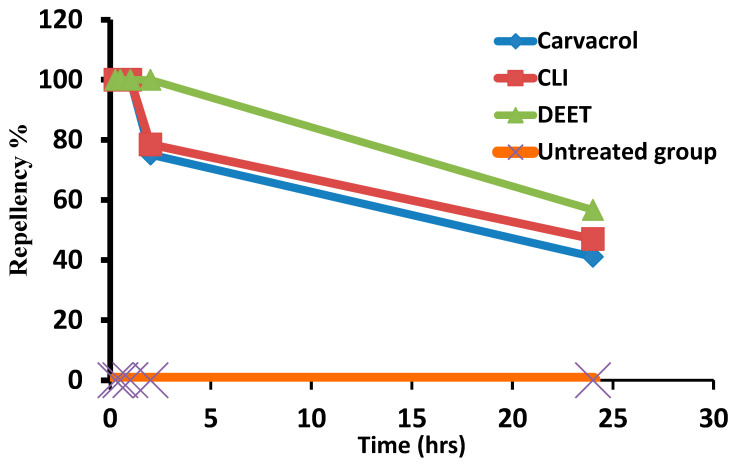
Repellency activity of carvacrol and carvacrol-loaded invasome (5%) forms using rod method against *R. annulatus* larvae.

Figure 5 illustrated the repellent activity of both types of carvacrol (5%) against *R. annulatus* larvae using the rod method. Both carvacrol and CLI showed repellent properties equivalent to that of the control positive (DEET 7%), particularly in the first hour after application. This repellent activity decreased over time, and after 2 h, it was significantly lower than DEET. The same observation was made after 24 h of treatment; however, CLI was nearly as effective as DEET at repelling (Figure 5).

### 3.9. Repellency Activity of Carvacrol-Loaded Invasome against Adult R. sanguineus Ticks

**Figure 6:**
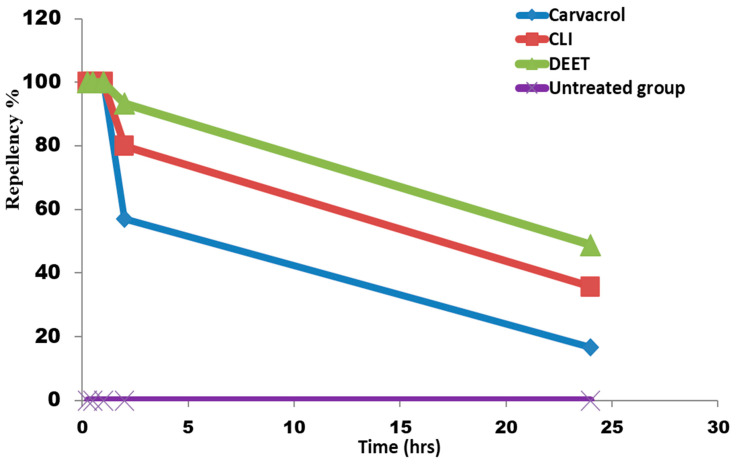
Repellency activity of carvacrol and carvacrol-loaded invasome (5%) using Petri-dish selective area choice method against *R. sanguineus* adult ticks.

When using the Petri dish selective area choice method, both forms of carvacrol had a repellent efficacy comparable to DEET within the first hour against adult *R. sanguineus* ticks. After 2 h, the repellent activity declined, and it was lower than that of DEET. After 24 h, the repellency percentage dropped dramatically for all treatments. However, CLI and DEET performed better than carvacrol (Figure 6, Supplementary Video S1).

### 3.10. Carvacrol/CLI Measurement in the Treated R. annulatus Adult Ticks by HPLC

**Figure 7:**
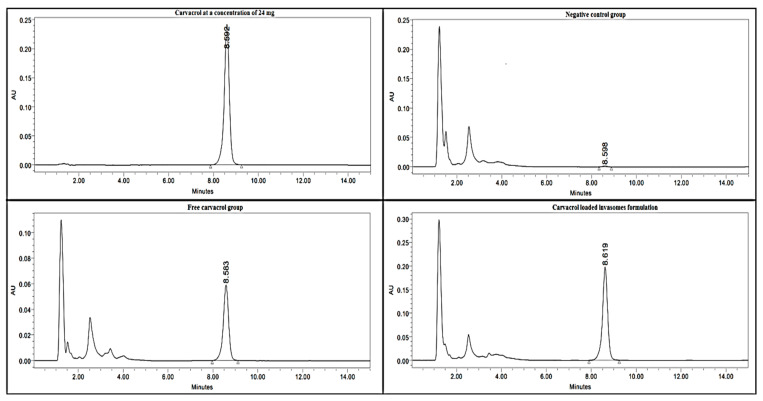
Peak area of CLI formulation and free carvacrol compared with that of carvacrol at a concentration of 24 mg.

**Figure 8:**
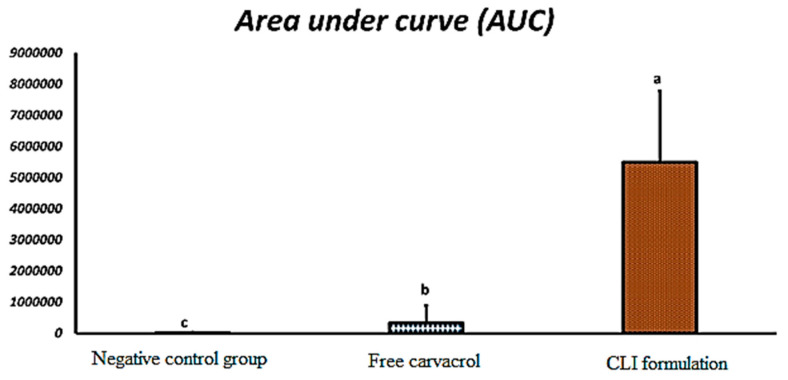
The permeation efficiency of CLI formulation inside the ticks’ cuticles compared with that of free carvacrol (mean + SE) with significantly difference at *p* < 0.001. a, b, and c means signficant difference.

Figure 7 depicts the peak area of different groups in comparison to that of carvacrol at a concentration of 24 mg. Ticks treated with CLI exhibited an insignificant (*p* > 0.001) peak area (3,777,677 ± 247,734 mAU·min) when compared to that of carvacrol at a concentration of 24 mg (3,825,411 ± 71,834 mAU·min). When compared to ticks treated with free carvacrol, ticks treated with CLI showed a significant (*p* < 0.001) increase in carvacrol permeation by 3.86 folds with significant Area Under the Curve (AUC) (Figure 8).

### 3.11. Antioxidants/Oxidants (GSH, MDA) and AchE Inhibition, in the Treated R. annulatus Adult Ticks

**Figure 9:**
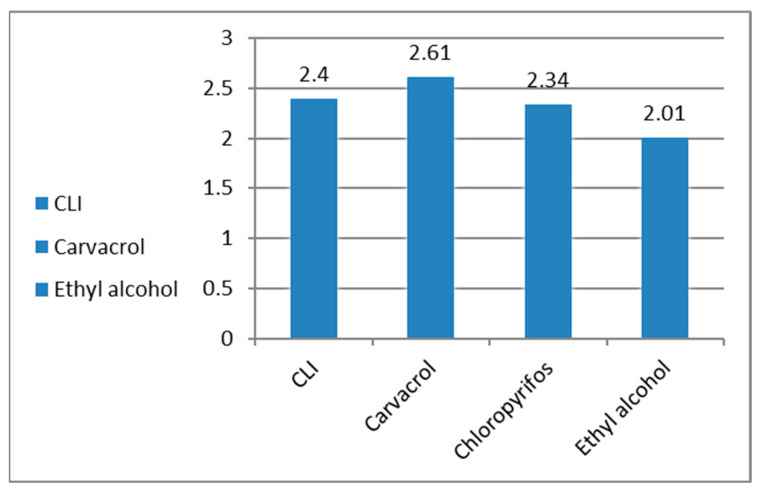
GSH level in the treated ticks by carvacrol and carvacrol-loaded invasomes.

**Figure 10:**
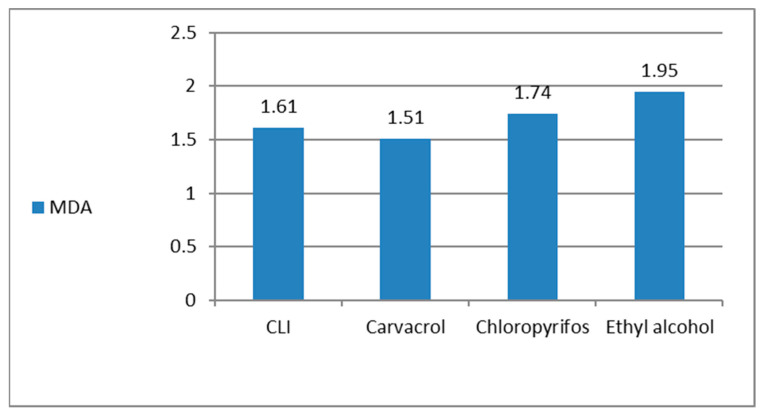
MDA level in the treated ticks by carvacrol and carvacrol-loaded invasomes.

**Figure 11:**
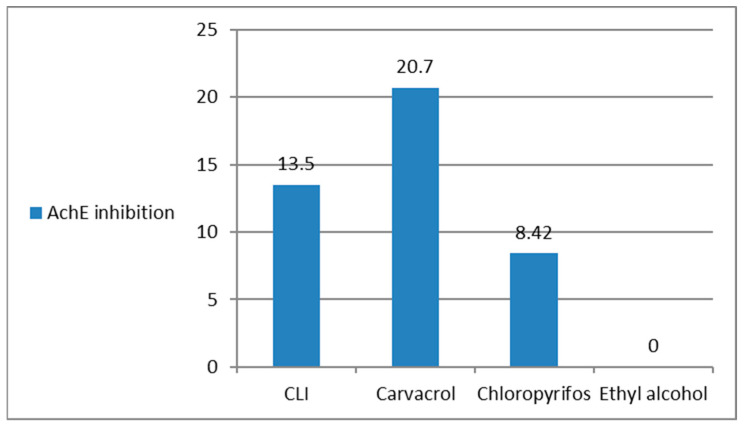
Acetylecholinestrase (AchE) inhibition percentage in the treated ticks by carvacrol and carvacrol-loaded invasomes.

GSH showed high activity in ticks treated with carvacrol or CLI when compared to the untreated control group (Figure 9). MDA levels were low in all treated groups due to mortality or acute toxicity of therapy (Figure 10). Ticks treated with pure carvacrol and CLI revealed an inhibition in AchE activity when compared to the control untreated ticks (Figure 11).

## 4. Discussion

Many new nanocarriers are being investigated in order to enhance drug permeability and effectiveness [42]. Among these nanocarriers, invasomes are a promising approach to improve the transdermal delivery and the permeability of drugs [22,43,44,45]. Invasomes are made up of phospholipids, cholesterol, ethanol, and terpenes [43,46]. Phospholipids serve as building blocks for lipid bilayers [47,48]. Cholesterol gives the lipid bilayer rigidity and stability [48]. Ethanol is a penetration promoter and a supplier of -ve charge [25]. Cineole is an effective terpene for drug delivery and improving transdermal flux into deep skin layers [43,44]. Preliminary investigations demonstrated that phospholipid and cholesterol have a synergistic effect on particle size and entrapment efficiency (EE%) [43]. Increasing the ethanol concentration above 3% resulted in a leaky and more fluidic lipid membrane, which allowed the entrapped drug to escape from the invasomes [25]. Therefore, the invasome synthesized in this study contained cineole (1% *v*/*v*), cholesterol (0.15% *w*/*w*), phospholipid (3% *w*/*w*), and ethanol (3% *v*/*v*). The standard calibration curve was shown to be reliable for quantifying carvacrol with a coefficient of determination (R2) of 0.999, indicating linearity. The presence of phospholipids and cholesterol increase the hydrophobicity and rigidity of the bilayer, resulting in fewer leaky and stable vesicles [43,45,49]. Furthermore, the presence of ethanol and terpenes in the invasomal lipid bilayer broke the hydrogen bonds between the ceramides in the phospholipid bilayer, increasing the available space for drug incorporation [46,50]. The size analysis revealed smaller particle sizes due to steric repulsion between terpene molecules [46,50]. Furthermore, ethanol reduces vesicle aggregation by increasing vesicle negative charges and electrostatic repulsion [25]. The polydispersity index analysis revealed a low polydispersity index, indicating the presence of homogeneity and a narrow particle size distribution. The negative surface charge of the CLI formulation indicated high physical and chemical stability, sufficient for electrostatic stabilization. Moreover, the DSC was used to show how the invasomes’ formulation affected the thermodynamic properties of their ingredients; in particular, that of carvacrol was affected [51,52] The absence of carvacrol’s thermal peak may be due to the high% EE of CLI and complete solubility of carvacrol. FT-IR spectra verified this finding by showing that the optimal CLI formulation had the same spectra as carvacrol, demonstrating the presence of carvacrol within the invasomes and the lack of interaction between the drug and the individual component of the invasomes.

The use of synthetic insecticides to control arthropod vectors has been gradually hampered by rapidly emerging vector resistance [53]. Therefore, alternative products are crucial for controlling ticks. This has offered up a substantial market for alternative products, such natural insecticides [54]. Natural insecticides from plants are readily biodegradable and do not leave any residues in the food or in the environment. Additionally, given to the various modes of action in which these substances work, it is unlikely that they will lead to the emergence of resistance [55,56]. In this context, the monoterpene carvacrol, which is the major ingredient of aromatic plants in the Lamiaceae and Verbenaceae families, is now being thought to be an effective control strategy due to its acaricide properties [57]. Carvacrol’s lipophilicity allows it to easily pass through the tick’s cuticle and into the tick’s body; it has the potential to damage the mitochondria and cell membrane at the cellular level [58]. It has the ability to interact with the cholinergic system as well [59].

In the present study, carvacrol and carvacrol-loaded invasomes (CLI) were tested at different concentrations against two tick species (*R. annulatus* and *R. sanguineus*). In the adult immersion test, the LC_50_ for carvacrol and CLI against *R. annulatus* were determined to be 4.30 and 2.60%, respectively. This finding is supported by the results of Pereira Junior et al. [60] as they reported LC_50_ of 20.11 mg/mL against *Rhipicephalus microplus* with product efficacy of 61.10%. Konig et al. [61] and Gonçalves et al. [62] also found that 4.5 µL and 5 µL/mL of acetyl carvacrol caused significant alterations in the reproductive indices of treated *Rhipicephalus microplus* due to remarkable morphological alterations in the female tick ovary.

Carvacrol and CLI both caused significant larval mortality in both tick species’ larvae (*R. annulatus* and *R. sanguineus*), with significant LC_50_ values (0.24 and 0.21%, and 0.27 and 0.23%, respectively). Lima et al. [63] investigated the activity of carvacrol against *R. sanguineus* and discovered that the integument surface of females exposed to the highest concentration (25 μL/mL) wrinkled, possibly due to dehydration or disruption of the integument’s cuticular and epithelial layers. It was recently found that, when carvacrol was encapsulated in yeast cell walls, it sustained its acaricidal action against *R. microplus* with an LC_50_ of 0.71 mg/mL compared to 1.82 mg/mL for carvacrol alone [64]. Carvacrol exhibited significant strong efficacy against *R. microplus* larvae, with LC_50_ of 0.22 mg/mL [65]. Furthermore, Pereira Junior et al. [60] assessed carvacrol activity against *R. microplus* at various concentrations (20, 40, 60, 80, and 100%) of the LC_50_, and it was discovered that the groups treated with 80 and 100% of the LC_50_ showed significant efficacy compared to the control group. Carvacrol was found to have significant acaricidal effect against *R. microplus* and *Dermacentor nitens* larvae, with 100% mortality at a low concentration (2.5 μL/mL) [66].

The repellency activity of carvacrol and CLI at 5% was tested against *R. annulatus* larvae using the rod method, and the results showed a significant repellency percentage after 2 h. However, there is no statistically significant difference between carvacrol repellency percentage and CLI. Furthermore, in comparison to the positive control treatment (DEET 7%), CLI demonstrated equivalent repellency to DEET. Additionally, the repellency activity of carvacrol and CLI against *R. sanguineus* adult ticks was tested using a petri-dish selective area choice method, and the results demonstrated a significant repellency percentage, even after 24 h. CLI displayed significantly greater repellency activity than pure carvacrol. Tick infestation can be effectively controlled by preventing tick adhesion to hosts through the use of repellent compounds [34,67,68]. Carvacrol and thymol displayed long-lasting repellant effects up to 14 days after spraying as well as toxic effects on *Dermanyssus gallinae* [69]. Similarly, Krober et al. [70] discovered that carvacrol had significant spatial repellency against mosquitos. Konig et al. [71] observed that when concentration increased, the repellency of carvacrol and acetylcarvacrol increased against adult *R. sanguineus* ticks. The repellent effect of carvacrol did not significantly increase with concentration, whereas acetylcarvacrol’s response was obviously dose-dependent (the maximum repellency index was recorded at 56 μL/mL) [71]. Carvacrol and thymol combinations also displayed over 90% repellency activity against *Ixodes ricinus* [72]. At 1-6 h post-treatment, nano-encapsulated carvacrol showed significant high repellency action against *R. microplus* larvae with a low repellency concentration value for 50% of the population (RC50) (0.13–0.27mg/cm^2^) [73]. Tick larvae can be disengaged from a host by unfavorable tactile stimulation, but a repellent can prevent tick larvae from adhering to a passing host [74]. In this study, CLI displayed a longer-lasting repelling effect when compared to pure carvacrol.

The oxidative stress and antioxidant activity of pure carvacrol and CLI were investigated. It was discovered that both forms induced oxidative stress on the treated ticks by elevating MDA and decreasing GST. In addition, carvacrol and CLI suppressed the activity of AchE in the treated ticks. These findings were supported by Aboelhadid et al. [75], who observed that carvacrol antioxidant activity plays a significant role in its acaricide activity. Cardoso et al. [76] also found that carvacrol inhibited the AChE of both the susceptible and resistant strains of *R. microplus*. Furthermore, Tavares et al. [18] reported that carvacrol and thymol reduced the activity of glutathione-S-transferase (GST), catalase (CAT), superoxide dismutase (SOD), and glutathione peroxidase (GPX) enzymes in the same tick population of *R. microplus*.

CLI surpassed pure carvacrol in terms of adulticidal and repellent properties. This result can be attributed to carvacrol passing through the cuticle of ticks; this result is supported by HPLC data that showed ticks treated with CLI formulation displayed a significant (*p* < 0.001) higher penetration than carvacrol by 3.86 folds. The presence of ethanol and terpene in the invasomal bilayer destroyed hydrogen connections between the ceramides in the tick’s cuticle, increasing the space available for medication incorporation [46,50]. Ethanol improves penetration and provides a -ve charge [25]. Cineole is an effective terpene for drug delivery and improving transdermal flux into deep skin layers [43,77].

This study has limitations in terms of the safety of carvacrol and CLI, although several works in the literature have reviewed the safety of carvacrol for both humans and animals [11,78]. In general, carvacrol is the safest chemical substance at low concentrations, has been approved by the FDA, and is utilized as a preservative in the food industry [11,79]. The effects of carvacrol on normal-healthy individuals were also examined in a clinical trial of Ghorani et al. [80], which established the carvacrol’s clinical safety and tolerability. In addition, the histological analysis of mice treated with vismodegib loaded invasomes revealed entirely normal skin structure, appearance, and architecture, with considerable improvement in all signs of the epidermis and dermis, confirming the safety of invasomes [27].

## 5. Conclusions

CLI achieved adulticidal and repellent effects better than pure carvacrol against *R. annulatus* and *R. sanguineus*, respectively. Invasomes increased carvacrol invasion to the tick cuticle. The results were approved by HPLC.

## Figures and Tables

**Table 1:** Adulticidal effect of carvacrol and carvacrol-loaded invasome against *R. annulatus* ticks.

Concentrations	Pure Carvacrol		Carvacrol-Loaded Invasome (CLI)	
	Mortality Rate	EPI	Mortality Rate	EPI
5%	62.0 ± 8.366 *	42.0 ± 1.295 *	100 ± 0.000 *	0.00 ± 0.000 *
2.5%	25.0 ± 5.477 *	51.7 ± 2.512 *	38.0 ± 8.366 *	41.7 ± 2.339 *
1.25%	0.00 ± 0.000	56.7 ± 4.096	20.0 ± 7.071 *	46.6 ± 2.972 *
0.625%	0.00 ± 0.000	66.1 ± 3.372	0.00 ± 0.000	66.8 ± 4.515
0.312%	0.00 ± 0.000	67.5 ± 4.520	0.00 ± 0.000	67.5 ± 4.520
0.156%	0.00 ± 0.000	65.9 ± 3.372	0.00 ± 0.000	66.7 ± 4.276
LC_50_	4.30%		2.60%	
LC_90_	6.31%		3.84%	
Distilled water	0.00 ± 0.000	67.1 ± 4.492	0.00 ± 0.000	67.1 ± 4.492
Ethyl alcohol 70%	0.00 ± 0.000	66.4 ± 3.003	0.00 ± 0.000	66.4 ± 3.003
Chlorpyrifos 25% (mL/L)	100 ± 0.000	0.00 ± 0.000	100 ± 0.000	0.00 ± 0.000

(*) significant for control negative. EPI= eggs production index.

**Table 2:** Larvicidal activity of carvacrol and carvacrol-loaded invasome against *R. annulatus* and *R. sanguineus* larvae.

Concentrations	Mortality of *R. annulatus* Larvae		Mortality of *R. sanguineus* Larvae	
	Pure Carvacrol	Carvacrol-Loaded Invasome (CLI)	Pure Carvacrol	Carvacrol-Loaded Invasome (CLI)
5%	100 ± 0.000 *	100 ± 0.000 *	100 ± 0.000 *	100 ± 0.000 *
2.50%	100 ± 0.000 *	100 ± 0.000 *	100 ± 0.000 *	100 ± 0.000 *
1.25%	100 ± 0.000 *	100 ± 0.000 *	100 ± 0.000 *	100 ± 0.000 *
0.63%	100 ± 0.000 *	100 ± 0.000 *	100 ± 0.000 *	100 ± 0.000 *
0.31%	73.2 ± 2.863 *	85.6 ± 3.507 *	63.2 ± 2.863 *	83.0 ± 3.391 *
0.16%	37.8 ± 1.923 *	42.4 ± 2.701 *	33.6 ± 3.209 *	36.8 ± 2.683 *
LC_50_	0.24%	0.21%	0.27%	0.23%
LC_90_	0.50%	0.48%	0.52%	0.49%
Distilled water	4.40 ± 1.140		5.00 ± 1.581	
Ethyl alcohol 70%	6.00 ± 1.581		5.40 ± 1.140	
Chlorpyrifos 25% (mL/L)	100 ± 0.000		100 ± 0.000	

(*) significant for control negative.

## Data Availability

All related data to this work are available in this manuscript.

## Supplementary Materials

The following supporting information can be downloaded at: https://www.mdpi.com/article/10.3390/microorganisms11030733/s1.
