# Supplementary figures and images for: Synthesis of Carvacrol-Loaded Invasomes Nanoparticles Improved Acaricide Efficacy, Cuticle Invasion and Inhibition of Acetylcholinestrase against Hard Ticks
Source: Microorganisms. 2023 Mar 13;11(3):733. doi: 10.3390/microorganisms11030733 (PMC10057972; doi:10.3390/microorganisms11030733)

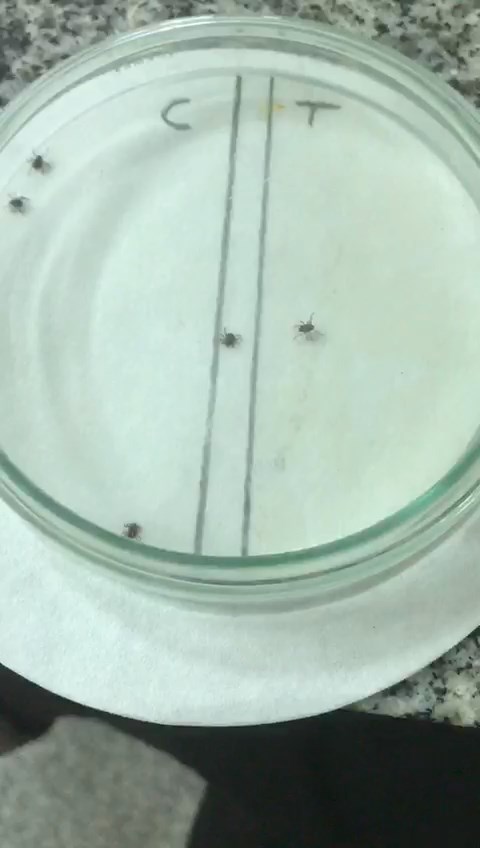

Supplement: Supplementary file 1 [file microorganisms-11-00733-s001.zip › microorganisms-2242356-Figure S1.jpg]
